# VERSE‐guided parallel RF excitations using dynamic field correction

**DOI:** 10.1002/nbm.3697

**Published:** 2017-02-17

**Authors:** Mustafa Çavuşoğlu, Ronald Mooiweer, Klaas P. Pruessmann, Shaihan J. Malik

**Affiliations:** ^1^ Institute for Biomedical EngineeringUniversity and ETH ZürichZürichSwitzerland; ^2^ Division of Imaging Sciences and Biomedical Engineering, King's College LondonSt. Thomas' HospitalLondonUK; ^3^ Center for Image SciencesUniversity Medical Center UtrechtUtrechtThe Netherlands

**Keywords:** excitation accuracy, field monitoring, gradient impulse response, parallel transmit, RF power, VERSE

## Abstract

In parallel RF pulse design, peak RF magnitudes and specific absorption rate levels are critical concerns in the hardware and safety limits. The variable rate selective excitation (VERSE) method is an efficient technique to limit the peak RF power by applying a local‐only RF and gradient waveform reshaping while retaining the on‐resonance profile. The accuracy of the excitation performed by the VERSEd RF and gradient waveforms strictly depends on the performance of the employed hardware. Any deviation from the nominal gradient fields as a result of frequency dependent system imperfections violates the VERSE condition similarly to off‐resonance effects, leading to significant excitation errors and the RF pulse not converging to the targeted peak RF power. Moreover, for iterative VERSE‐guided RF pulse design (i.e. reVERSE), the *k*‐space trajectory actually changes at every iteration, which is assumed to be constant. In this work, we show both theoretically and experimentally the effect of gradient system imperfections on iteratively VERSEd parallel RF excitations. In order to improve the excitation accuracy besides limiting the RF power below certain thresholds, we propose to integrate gradient field monitoring or gradient impulse response function (GIRF) estimations of the actual gradient fields into the RF pulse design problem. A third‐order dynamic field camera comprising a set of NMR field sensors and GIRFs was used to measure or estimate the actual gradient waveforms that are involved in the VERSE algorithm respectively. The deviating and variable *k*‐space is counteracted at each iteration of the VERSE‐guided iterative RF pulse design. The proposed approaches are demonstrated for accelerated multiple‐channel spatially selective RF pulses, and highly improved experimental performance was achieved at both 3 T and 7 T.

**Abbreviations**
AFI: actual flip‐angle imaging
GIRF: gradient impulse response function
NRMSE: normalized root mean square error
SAR: specific absorption rate
VERSE: variable rate selective excitation

## 1 INTRODUCTION

Beyond typical imaging experiments, where excitation of sharp slice profiles is desired, RF pulses with spatial selectivity in multiple dimensions are primarily used to shape the spatial flip‐angle distribution.^1^ However, the practical application of tailored RF pulses is generally hampered by long pulse durations. Parallel RF transmission has emerged as a powerful technology to shorten pulse durations and control the RF power, at the cost of being more prone to high RF power and specific absorption rate (SAR).^2^, ^3^ Furthermore, high acceleration factors in parallel RF transmission critically push the RF power demand.^4^ Recently, several sophisticated RF pulse design algorithms under strict power and SAR constraints^5^, ^6^ have been proposed to circumvent this issue.

A simple alternative to tackle such an RF power control problem is the variable rate selective excitation (VERSE) method, which uses local reshaping of RF pulses and gradient waveforms.^7^, ^8^, ^9^, ^10^, ^11^ Because the RF power constraint is handled by applying a variable rate stretching or shrinking to the gradient design problem, VERSE is a faster method than constrained numerical optimization by altering the gradient waveforms directly. The key condition in VERSE method is to maintain for each sample the RF‐to‐gradient amplitude ratio that preserves the rotational behavior of on‐resonance spins.^8^ However, this condition can be easily violated, because the VERSE reshaping does not take into account the off‐resonances, which could notably modulate the spin rotations.^12^ To compensate for such additional spin behavior terms to retain the VERSE condition, the ability of numerical RF pulse design methods to make off‐resonance corrections have recently been adapted to variable rate selective excitation iteratively with a peak RF power constraint (reVERSE method).^12^, ^13^

Experimentally, the maintenance of VERSE conditioning strictly depends on the performance of the gradient system, implying that any deviation from the nominal gradient waveforms will disrupt the local field similarly to off‐resonances and hence the RF‐to‐gradient ratio, ultimately resulting in excitation errors. In practice, actual gradient waveforms are prone to deviations from their ideal counterparts primarily due to the physical limits of the employed hardware such as induced eddy currents,^14^, ^15^ bandwidth limitations of the amplifiers, mechanical vibrations at gradient switching^16^ and thermal variations.^17^ Such imperfections mean that, for iterative parallel RF pulse design methods such as reVERSE, the *k*‐space trajectory—which is nominally held constant—actually changes at every iteration. Therefore, in the context of parallel excitation, many efforts have been made to counteract for the excitation errors due to the *k*‐space deviations exploiting either image‐based estimations of the actual gradient waveforms^18^, ^19^, ^20^ or model‐based estimations of the effects of one component of the error terms, such as eddy currents in the RF pulse design.^21^

A generic approach is to treat the gradients as a linear time‐invariant system, and hence characterize their response via a gradient system impulse response function (GIRF), which may be used within pre‐compensation and post‐correction methods.^22^, ^23^, ^24^, ^25^ Once GIRF of the system is accurately measured or calculated, it can be used as a calibration tool before each RF pulse design, providing a fast estimate of the actual gradient field.

A recent advancement to obtain the actual gradient waveforms played out by the scanner with a very high accuracy is the ability of concurrent field monitoring using NMR field probe arrays.^26^, ^27^, ^28^ Such a measurement directly captures the dynamics of all externally induced field perturbations that affect the spin evolution. Gradient field measurements using field probes have been recently exploited to calculate the GIRFs with sufficient bandwidth and frequency resolution,^25^, ^29^ retrospective correction of physiological field fluctuations in high‐field brain imaging,^30^ real‐time field stabilization 31 and improving the excitation accuracy in parallel RF transmission.^32^

In this work, we show both theoretically and experimentally that the performance of the reVERSE method is strictly dependent on the gradient system fidelity. To push the excitation accuracy, besides limiting the RF power below certain thresholds by preserving the VERSE condition for every reVERSE iteration, we propose to integrate gradient field monitoring using a dynamic field camera or GIRF estimations of the actual gradient fields into the RF pulse design problem. Addressing the deviating and variable *k*‐space challenges in the existing reVERSE method through both of the proposed approaches provided highly improved experimental performance at 3 T and 7 T. With both of the proposed methods, any gradient waveform can be dynamically corrected without requiring any analytical description.

## 2 THEORY

### 2.1 VERSE and reVERSE principle

VERSE is a method to limit the peak RF power by solving a simpler gradient optimization problem rather than an RF pulse design problem by dynamically dilating the gradient waveforms and associated RF pulse by traversing the initial excitation *k*‐space trajectory at different speed rates such that the rotational behavior of on resonance spins is preserved.^8^ The principal determinant of keeping the spin rotation unchanged is to maintain the RF‐to‐gradient amplitude ratio in excitation *k*‐space. Several approaches have been reported in the literature to fulfill the VERSE condition, predominantly using a time‐dilation function *τ*(*t*) to scale the original waveform pair {*B*_1_(*t*), **G**(*t*)}, where *B*_1_(*t*) = *B*_1 , *x*_(*t*) + i*B*_1 , *y*_(*t*) is the time‐varying complex‐valued RF pulse envelope and **G**(*t*) = [*G*_***x***_(*t*), *G*_***y***_(*t*), *G*_***z***_(*t*)]^T^ includes the gradient waveforms designed to generate the excitation *k*‐space trajectory respecting the hardware limits. Throughout the paper *B*_1_ is used to represent the envelope of the complex RF pulse and $B_{1}^{+}$ is used to represent the complex transmit field. In this work we used a recent implementation which rules out the use and optimization of *τ*(*t*) by transforming the original waveform pair {*B*_1_(*t*), **G**(*t*)} into the Euclidian arc‐length *s*‐domain {*B*_1_(*s*), **G**(*s*)}, where *s* measures the total distance traversed in the excitation *k*‐space^33^ such that

(1) $$\begin{array}{l} s\left(t\right)\equiv \gamma \int_{0}^{t}\left|G\left(\tau \right)\right|d\tau \\ B_{1}\left(s\left(t\right)\right)=B_{1}\left(t\right)\\ G\left(s\left(t\right)\right)=G\left(t\right) \end{array}$$

In the *s*‐domain, the peak RF constraint is equivalently translated to the maximum gradient amplitude constraint.^13^ The VERSE condition including the variably stretched waveform pair $\left\{B_{1}^{v}\left(s\right),G^{v}\left(s\right)\right\}$ is expressed as

(2) $$\frac{B_{1}\left(s\right)}{G\left(s\right)}=\frac{B_{1}^{v}\left(s\right)}{G^{v}\left(s\right)}\equiv W\left(s\right)$$

where *W*(*s*) represents an invariant RF‐to‐gradient amplitude ratio, which implies that the RF‐to‐gradient ratio is invariant at every arc‐length. Then the peak RF constraint is linked to the gradient amplitude constraint such that

(3) $$\left|B_{1}^{v}\left(s\right)\right|\leq B_{1,\max}\Leftrightarrow \left|G^{v}\left(s\right)\right|\leq \frac{B_{1,\max}}{W\left(s\right)}$$

In order to retain respect of the hardware limits, the VERSE gradients must satisfy for all *s*

(4) $$\left|G^{v}\left(s\right)\right|\leq \min\left\{\frac{B_{1,\max}}{W\left(s\right)},G_{\max}\right\}$$

where *B*_1, max_ and *G*_max_ correspond to the RF and gradient limits respectively. This approach has the advantage of providing time‐optimal waveform pairs and guaranteeing identical spin rotations in RF excitation schemes. Besides taking into account the off‐resonance effects, the reVERSE algorithm^12^ iteratively reduces the peak RF magnitude, where complex‐valued transverse magnetization *M*_*xy*_ is linked to the complex‐valued RF pulse *b*_*c*_(*t*) of channel *c* in the small‐tip angle regime as

(5) $$M_{\mathrm{xy}}\left(r,T_{0}\right)=i\gamma M_{0}\left(r\right)\sum_{c}B_{1}^{c}\left(r\right)\int_{0}^{T_{0}}e^{ik\left(t\right).r}e^{\left(i\Delta \omega \left(r\right)\right)\left(t-T_{0}\right)}b_{c}\left(t\right)\mathrm{dt}$$

(6) $$k\left(t\right)=-\gamma \int_{t}^{T_{0}}G\left(t^{\prime }\right)dt^{\prime }$$

where **r** denotes the position in space, *t* is time, *T*_0_ is the total pulse duration, *γ* is the gyromagnetic ratio of the nucleus of interest, Δ*ω* = *γ*Δ*B*_0_(**r**) is the local off‐resonance frequency and $B_{1}^{c}\left(r\right)$ is the complex‐valued transmit sensitivity map of coil *c*.

### 2.2 Effects of gradient system imperfections and RF pulse design

From the *s*‐domain perspective, the incremental spin rotation is described by solving the Bloch equations (neglecting the relaxation terms):

(7) $$\begin{array}{l} \phi^{\prime }\left(s,r\right)=-\sqrt{{\left|W\left(s\right)\right|}^{2}+{\left(g\left(s\right).r\right)}^{2}}\;\\ n\left(s,r\right)\propto \left(\frac{B_{1,x}\left(s\right)}{\left|G\left(s\right)\right|},\frac{B_{1,y}\left(s\right)}{\left|G\left(s\right)\right|},g\left(s\right).r\right) \end{array}$$

where *ϕ*^′^(*s*, **r**) is the incremental rotation angle about the axis of rotation **n**(*s*, **r**), **g**(*s*) is the unit gradient field vector and *W*(*s*) is the ratio of the RF and gradient amplitude.^13^, ^34^

The iterative reVERSE method is based on the assumption that the *k*‐space trajectory associated with the RF pulse design problem is unchanged at each iteration. However, due to the non‐ideal performance of the gradient system, it is required to make a distinction between the nominal gradient waveforms **G**_nom_ and the actual gradient fields experienced by the sample inside the scanner **G**_act_. The deviations from the nominal gradient fields locally alter the incremental spin rotation, which ultimately results in the violation of the VERSE condition, similar to off‐resonance effects (i.e. $e^{\left(i\text{Δ}\omega \left(r\right)\right)\left(t-T_{0}\right)}$ term in Equation (5)). To account for such time‐varying local gradient field perturbations, Equation (7) has to be modified by an additional gradient field deviation term $\tilde{G}\left(s\right)$:

(8) $$\begin{array}{l} \phi^{\prime }\left(s,r\right)=-\sqrt{{\left|W\left(s\right)\right|}^{2}+\left(g\left(s\right).r+\frac{\tilde{G}\left(s\right).r+\Delta \omega \left(r\right)}{\gamma \left|G_{\mathrm{nom}}\left(s\right)\right|}\right)}\\ n\left(s,r\right)\propto \left(\frac{B_{1,x}\left(s\right)}{\left|G_{\mathrm{nom}}\left(s\right)\right|},\frac{B_{1,y}\left(s\right)}{\left|G_{\mathrm{nom}}\left(s\right)\right|},g\left(s\right).r+\frac{\tilde{G}\left(s\right).r+\Delta \omega \left(r\right)}{\gamma \left|G_{\mathrm{nom}}\left(s\right)\right|}\right)\\ \tilde{G}\left(s\right)=G_{\mathrm{nom}}\left(s\right)-G_{\mathrm{act}}\left(s\right) \end{array}$$

**Figure 1:**
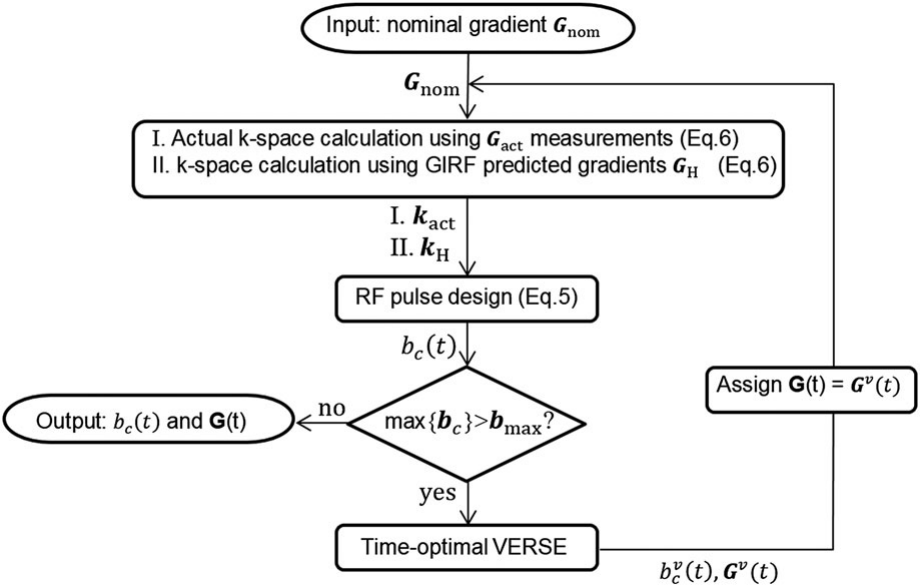
Flow‐chart of modified reVERSE RF pulse design algorithm including the *k*‐space trajectory monitoring and estimation proving the knowledge of the actual *k*‐space trajectories

Our approach to minimize the degradation of the VERSE condition due to the gradient field perturbations is to measure all the relevant system and experimental parameters and integrate the error terms into the pulse design algorithm. Such measurements using arrays of NMR‐based field probes allow monitoring of the spatio‐temporal dynamics of fields in single‐shot measurements with exquisite precision and high bandwidth. Furthermore, with the assumption that the gradient chain behaves as a linear time‐invariant system, it is possible to determine the GIRF of the system as a one‐time calibration procedure, based on which the actual gradient waveforms can be predicted very fast with a certain accuracy. Figure 1 illustrates the modified reVERSE pulse design algorithm taking into account the effects described in Equation (8).

The RF pulses are designed based on the actual *k*‐space trajectory **k**_act_ (actual *k*‐space trajectory calculated from the actual gradient waveforms directly measured by a field camera) and/or predicted *k*‐space trajectory **k**_H_ (predicted *k*‐space trajectory calculated from the gradient waveforms estimated using the GIRF approach). If the resulting peak RF amplitude exceeds the given limits then the variably stretched gradient waveforms ***G***^*v*^(*t*) are calculated by using the time‐optimal VERSE method and applied as the input for the next iteration of the algorithm. Note that an attenuation factor *α* = 0.95 is applied to set a slightly lower amplitude constraint on the time‐optimal VERSE procedure than the target amplitude constraint to improve the convergence behavior of the peak *B*_1_ amplitude against small oscillatory overshoots.^12^

## 3 MATERIALS AND METHODS

MRI measurements were made using two separate setups. Setup A: Philips Achieva 3 T (Philips Healthcare, Best, The Netherlands) fitted with an eight‐channel parallel transmit body coil^35^ with maximum gradient amplitude of 40 mT/m and slew rate of 200 T/m/s. An eight‐channel receiver head coil was used in experiments. Setup B: Philips Achieva 7 T whole‐body MR system (Philips Healthcare, Cleveland, OH, USA) equipped with an eight‐channel parallel transmit system (Philips Research Laboratories, Hamburg, Germany) each powered with 1 kW RF amplifier, with maximum gradient amplitude of 40 mT/m and slew rate of 200 T/m/s. An eight‐channel shielded loop transmit array (Rapid Biomedical, Rimpar, Germany) was used for multichannel RF transmission with an inbuilt transmit/receive switch. Signal was received with a 16‐channel receive array (Nova Medical, Wilmington, MA, USA) inserted into the transmit array operated in the circularly polarized mode.

### 3.1 Field monitoring

Gradient field measurements were performed with a third‐order dynamic field camera comprising a 16‐channel acquisition system in addition to the transmission and receive chains to operate a set of NMR field sensors.^26^, ^27^ The field probes, with a signal lifetime of about 40 ms, were evenly distributed on the surface of a sphere, and their positions in the scanner were determined by measuring NMR frequency shifts under static gradients of 2.5 mT/m in the *x*, *y*, *z* directions. Subsequent data processing includes routing the probe signal by means of transmit/receive switches to receive chains (preamplification, analog filtering, second amplifier stages), sampling and digital conversion to 1 MHz output bandwidth by a custom‐configured spectrometer based on high‐speed analog‐to‐digital converters (14 bit, 250 MS/s) and field programmable gate arrays (FPGA, National Instruments, Austin, TX, USA). The phase of the NMR signal at each probe is proportional to the time‐integral of the magnetic field magnitude at the probe position. The field inside the object was directly interpolated using the computed probe position and the obtained field measurement.^27^

### 3.2 Gradient impulse response measurements

An alternative approach to characterize the dynamic performance of a gradient system is based on the measurements of the GIRF. Such an approach relies on the assumption that the system is largely linear and time invariant. In theory, to achieve an estimation of GIRF, the Fourier transform of the system input should ideally be equal at all frequencies of interest, requiring a broadband pulse with a flat spectral energy distribution. Due to the physical limits of applicable input amplitude of delta‐like functions in the time domain, we instead used linear frequency‐swept pulses (chirps) as the gradient waveforms to successively encode the different frequencies in the bandwidth and applied four chirp input pulses with bandwidths of 10, 20, 30 and 40 kHz and durations about 10 ms. Among the real‐valued spherical harmonics spanning the field, the zeroth‐order function represents the average field, whereas the three first‐order harmonics correspond to the linear gradients in the *x*, *y* and *z* directions. Note that standard pre‐emphasis of the gradient system for eddy current compensation was enabled for all experiments. The GIRF measurements in Setup B were made using the same dynamic field camera and associated hardware as described above for gradient field monitoring, and GIRFs were calculated from multiple measurements using Equation (5) in Reference 29. For each of the response measurements, probe signals were acquired with a duration of 70 ms, providing a frequency resolution of about 14.3 Hz. The whole measurement procedure was repeated separately for the *x*, *y* and *z* gradient channels. In Setup A, GIRF measurements were made using chirped test waveforms with an image‐based gradient estimation technique.^36^ Due to practical limitations, chirp waveforms of duration 6.4 ms were used, giving a frequency resolution of 156 Hz. In Setup B, the GIRF predicted gradient waveforms were compared with the corresponding direct measurements for verification.

### 3.3 Phantom experiments and parallel excitation

A 16 cm diameter spherical flask phantom containing CuSO_4_ solution (*T*_1_ ~ 270 ms) and a 10 cm diameter spherical saline phantom containing 100 mM sodium chloride solution (*T*_1_ ~ 270 ms) was used for measurements in Setup A and Setup B respectively. $B_{1}^{+}$ quadrature mode of the transmit array was obtained to achieve a constructive superposition of the individual channels by setting the phase values appropriately. Complex‐valued $B_{1}^{+}$ maps were obtained for each transmit channel by using actual flip‐angle imaging (AFI)^37^ with the following acquisition parameters: *T*_E_ = 2.8 ms, FOV = 128 × 128 mm^2^, slice thickness = 4 mm, flip angle =60°, *T*_R1_/*T*_R2_ = 40/200 ms, matrix size =64 × 64 × 3. Data was acquired using a 3D acquisition with three slices, with the central slice used for RF pulse design, in order to avoid slice profile effects.^38^ Additionally, Δ*B*_0_ maps were estimated using two gradient‐echo acquisitions at *T*_E1_/*T*_E2_ = 5/6 ms to compensate for the off‐resonance in the RF pulse design. The obtained $B_{1}^{+}$ and Δ*B*_0_ maps were incorporated for the RF pulse design for both numerical simulations and scanner experiments.

**Figure 2:**
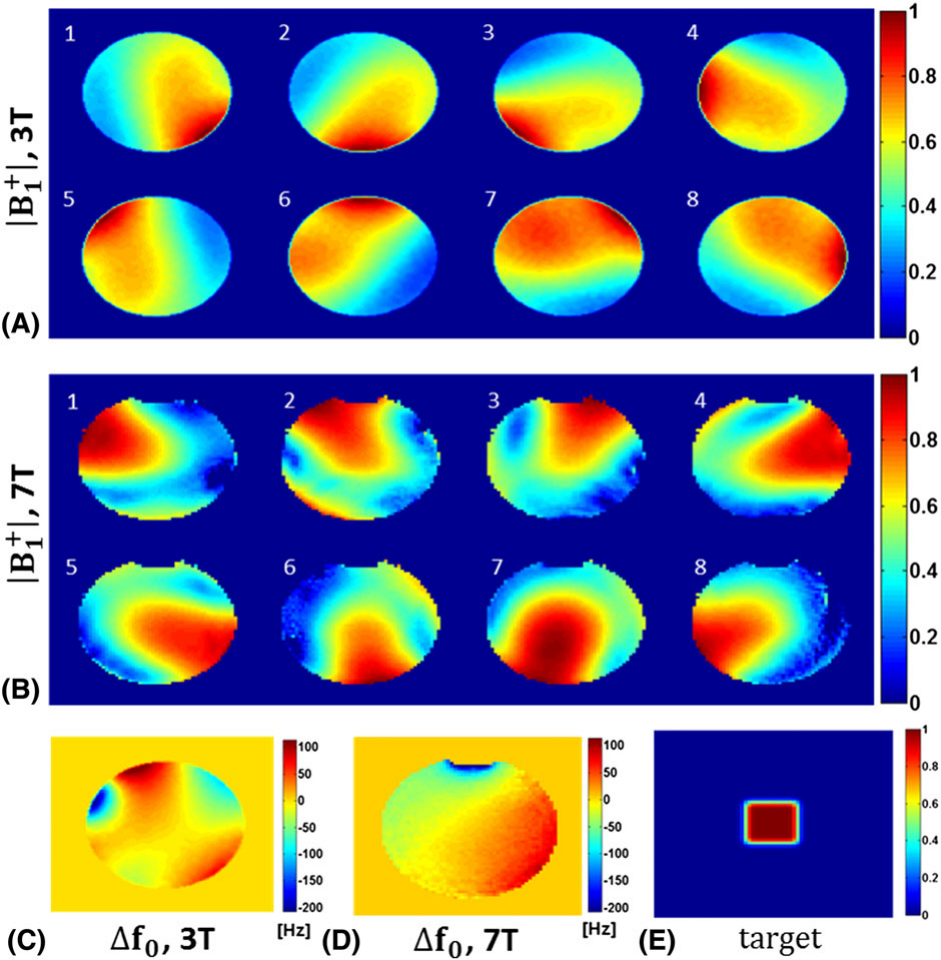
All spatial‐domain information for 2D spatially selective excitation. A, Normalized $\left|B_{1}^{+}\right|$ maps of an eight‐channel body transmit system of a 3 T scanner. B, Normalized $\;\left|B_{1}^{+}\right|$ maps of an eight‐channel transmit head array operating at 7 T. C, Static off‐resonance map at 3 T. D, Static Off‐resonance map at 7 T. E, Target excitation pattern

Initial 2D spatially selective multichannel RF pulses were designed based on the spatial domain method of Grissom et al.^39^ using a conjugate gradient algorithm along with the L‐curve criterion. The desired excitation pattern was a 30 × 30 mm^2^ square sampled on a 64 × 64 Cartesian grid in a 128 × 128 mm^2^ region. The rectangular target profile was blurred by applying a Gaussian convolution kernel of 1 cm full‐width at half‐maximum to cancel out the aliased excitations at the edges. Figure 2 shows all spatial‐domain information for 2D spatial excitation in both Setup A and Setup B.

In all cases a single‐shot spiral‐in excitation *k*‐space trajectory was designed using time‐optimal gradient waveforms with maximum gradient amplitude of 30 mT/m and maximum slew rate of 180 T/m/s and encoding a 128 mm FOV with 2 mm resolution and radially undersampled to accelerate (2×) the excitation. The RF and gradient waveforms were sampled with a 6.4 μs dwell time for scanner implementations. Gradient ramps were appended at the beginning of the designed gradient waveforms if they start from a non‐zero value and RF pulses were set to be zero during this time. All computations including RF pulses and gradient designs and numerical simulations were performed in MATLAB (MathWorks, Natick, MA, USA).

We performed parallel RF excitation experiments in both Setup A and Setup B by acquiring axial images with a single‐shot turbo spin echo sequence with the following parameters: FOV = 128 × 128 mm^2^; *T*_R_/*T*_E_ = 2500/250 ms; matrix size =96 × 96. Multichannel RF pulses and gradient waveforms were time aligned with a sub‐dwell precision of 1 μs and set accurately for all experiments.^40^

We first estimated the actual gradient waveforms played out by the scanner via time domain convolution of the nominal waveforms with GIRF computed using the above explained image domain method in Setup A. The RF pulses were designed to achieve 90° flip angle. This initial RF and gradient waveform pair were applied to the algorithm (Figure 1) to calculate the reVERSE pulses by redesigning the RF pulses based on the estimated actual *k*‐space trajectories at every iteration for a target maximum peak RF magnitude of 12 μT (~74% reduction relative to the initial peak RF magnitude).

We further obtained the actual gradient waveforms at every iteration of the algorithm by direct field camera measurements in Setup B. Initial RF pulses were designed based on the measured *k*‐space trajectories with the target peak RF magnitude of 12 μT (50% reduction relative to the initial peak RF magnitude). The attenuation factor *α* is set to 0.95 for all experiments to speed up the convergence. We additionally repeat all the experiments in Setup B with the same settings for comparison using the actual gradient waveforms predicted by GIRFs that are computed using the field camera measurements. Matlab source code can be downloaded from https://github.com/mriphysics/reverse‐GIRF.

### 3.4 *In vivo* parallel excitation

A volunteer study was performed to demonstrate the potential benefits of the proposed methods *in vivo* at 3 T using the same parallel transmit system as described for Setup A. Experiments were performed on a single healthy adult male volunteer; ethical approval was obtained for the *in vivo* scan and the volunteer supplied written consent. As an example, high flip‐angle inhomogeneity mitigation was selected, which is a common practical application of parallel excitation. Multichannel RF pulses were designed for the ‘spiral non‐selective’ (SPINS) *k*‐space trajectory for whole brain excitation,^41^ with target flip angle 90°. Whole head 3D $B_{1}^{+}$ maps were acquired using a combination of a quadrature‐mode AFI^37^ acquisition (*T*_E_ = 0.74 ms, flip angle 80°, *T*_R1_/*T*_R2_ = 30/150 ms) and low flip angle spoiled gradient echo images (flip angle 1°) using the interferometric approach.^42^ A 3D Δ*B*_0_ map was also acquired using two gradient‐echo acquisitions at *T*_E1_/*T*_E2_ = 2.3 and 4.6 ms. All $B_{1}^{+}$/*B*_0_ mapping acquisitions used FOV = 260 × 250 × 165 mm^3^ and matrix =52 × 50 × 37. The FSL brain extraction tool (BET) was used to create a mask for the whole brain in 3D.^43^ SPINS pulses were designed to produce a 90° excitation within this mask. reVERSEd pulses were calculated by redesigning the initial RF pulses based on the GIRF estimated *k*‐space trajectories at every iteration for a target maximum peak RF magnitude of 13.5 μT (~70% reduction relative to the initial peak RF magnitude). The quality of the resulting excitation was measured by using the resulting SPINS pulse as the excitation pulse in an AFI $B_{1}^{+}$ mapping scan, to directly measure the resulting flip angle using the same scan parameters described above. For comparison, a standard hard pulse excitation was performed using the quadrature mode of the eight‐channel transmit coil for a nominal flip angle of 90°.

## 4 RESULTS

**Figure 3:**
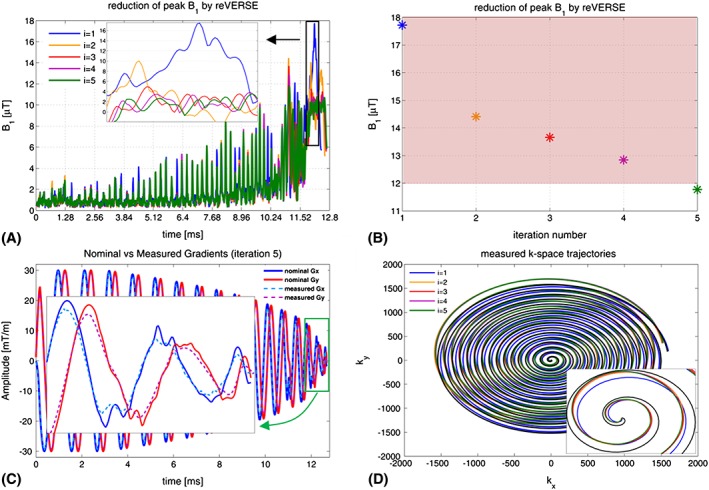
A, RF waveforms at each reVERSE iteration. B, Reduction of peak RF power by iterative reVERSE algorithm. C, Gradient waveform deviations (Setup B). D, *k*‐space trajectory deviations at each reVERSE iteration, which are supposed to be the same

Figure 3 depicts the existing gradient non‐idealities and associated *k*‐space deviations in reVERSE pulse design method obtained in Setup B. By applying the reVERSE method, the peak RF magnitude was gradually reduced below the target magnitude (12 μT) in five iterations. Figure 3A shows the individual RF waveforms and Figure 3B shows the corresponding peak RF values at each iteration. However, both the magnitude and pattern of the actual gradient fields significantly deviate from their nominal counterparts, which are shown here as the resulting reVERSEd gradient waveforms at the final iteration (Figure 3C). Actual gradient strengths were decreased relative to the peak values of the nominal waveforms. The final pattern of the gradients as a result of VERSE stretching was substantially smoothed due to the well‐known low‐pass characteristics of the gradient system. Figure 3D illustrates the discrepancy of the associated *k*‐space trajectories iteration to iteration, which is assumed to be unchanged by the reVERSE algorithm.

**Figure 4:**
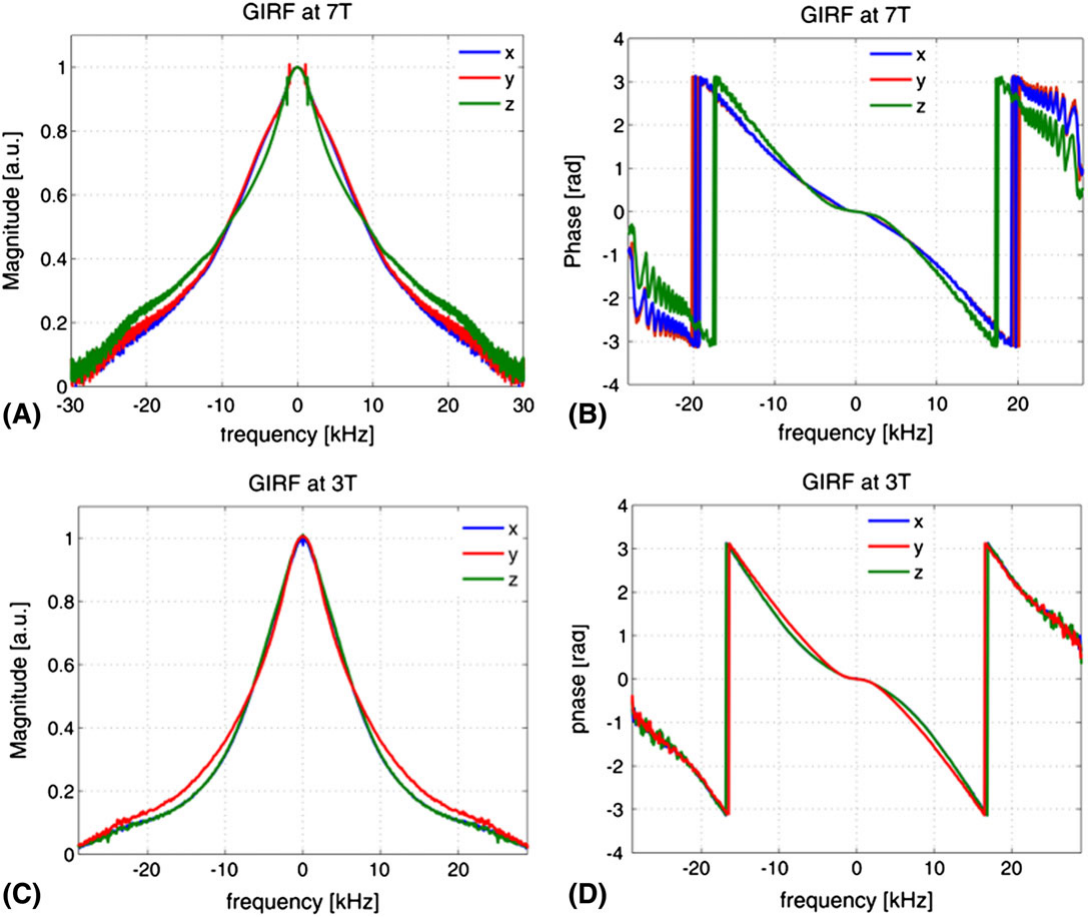
Measured GIRFs in frequency domain for all gradient axes; the frequency resolution was 100 Hz, dictated by the duration of the test waveforms (10 ms). The *x* and *y* axes are very similar, while the *z* performance is slightly different

Magnitude and phase plots of the GIRFs for all three gradient directions, which are measured using a dynamic field camera at 7 T and image‐based computations at 3 T, are shown in Figure 4. The low‐pass characteristic of the gradient system is apparent for both systems, where the eddy current compensation presumably broadens the response plateau at low frequencies. A further common feature of both systems is that *x* and *y* gradient axes exhibit similar responses (to be expected as they are similar designs), while the *z* gradient has a slightly narrower bandwidth at half maximum. The flat phase patterns observed around DC (zero frequency) in Figure 4B and Figure 4D imply almost‐zero net delay in all gradient channels, which reflects an appropriate delay calibration. Note that, with the higher frequency corresponding to lower input power, the noise in the GIRF waveforms increases, which is more pronounced with the measured GIRFs in the 7 T system in both magnitude and phase plots. The magnitude plots of GIRFs at 7 T exhibit channel‐specific patterns of several distinct peaks in the low‐frequency range of 600–1800 Hz, which most likely correspond to the mechanical resonances of the gradient coils. These resonances and their frequencies are in good agreement with previously reported acoustic responses of the gradient system.^25^, ^44^

**Figure 5:**
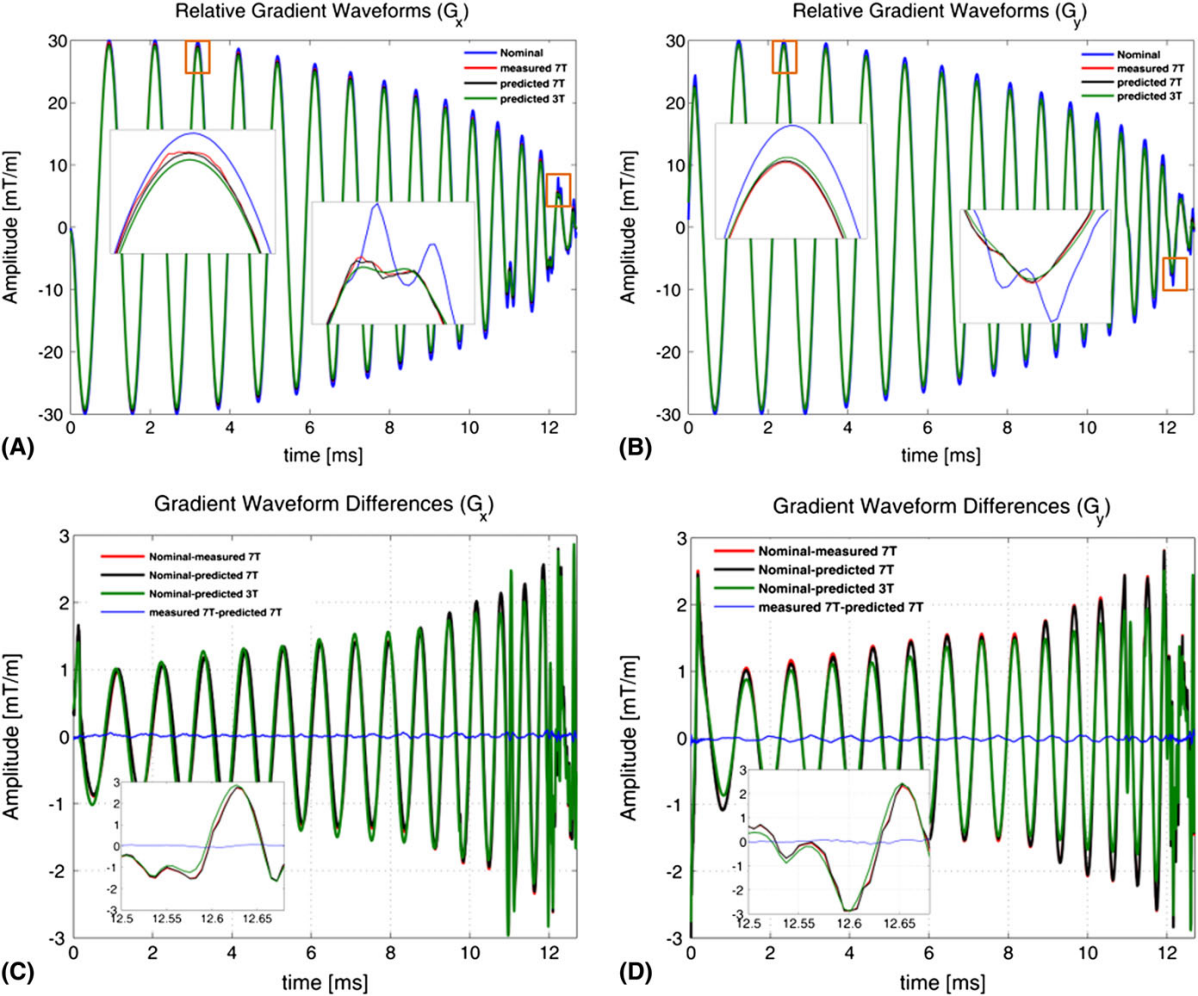
Comparison of nominal, GIRF predicted and monitored reVERSE gradients and relative differences

Figure 5A,B provides a comparison between the nominal, measured and GIRF predicted reVERSEd gradient waveforms in the *x* and *y* directions. The zoomed details from the red frames better illustrate the gradient field deviations from the nominal waveforms, where the substantial magnitude and waveform pattern discrepancy is apparent. The measured and predicted gradients at 7 T are highly similar. Predicted waveforms at 3 T are slightly different because this system has a physically different response. Figure 5C,D plots the relative differences of all gradient waveforms. The deviation from the nominal gradient waveforms is around 3% in magnitude and increases over time to 10%. The difference between the directly measured and GIRF predicted gradient waveforms at 7 T oscillates around the zero‐line (better visible in the zoomed frames) with absolute maximum amplitude of 0.11 mT/m in the *x* direction and 0.12 mT/m in the *y* direction, implying that the GIRF predicted and measured gradient waveforms agree closely.

**Figure 6:**
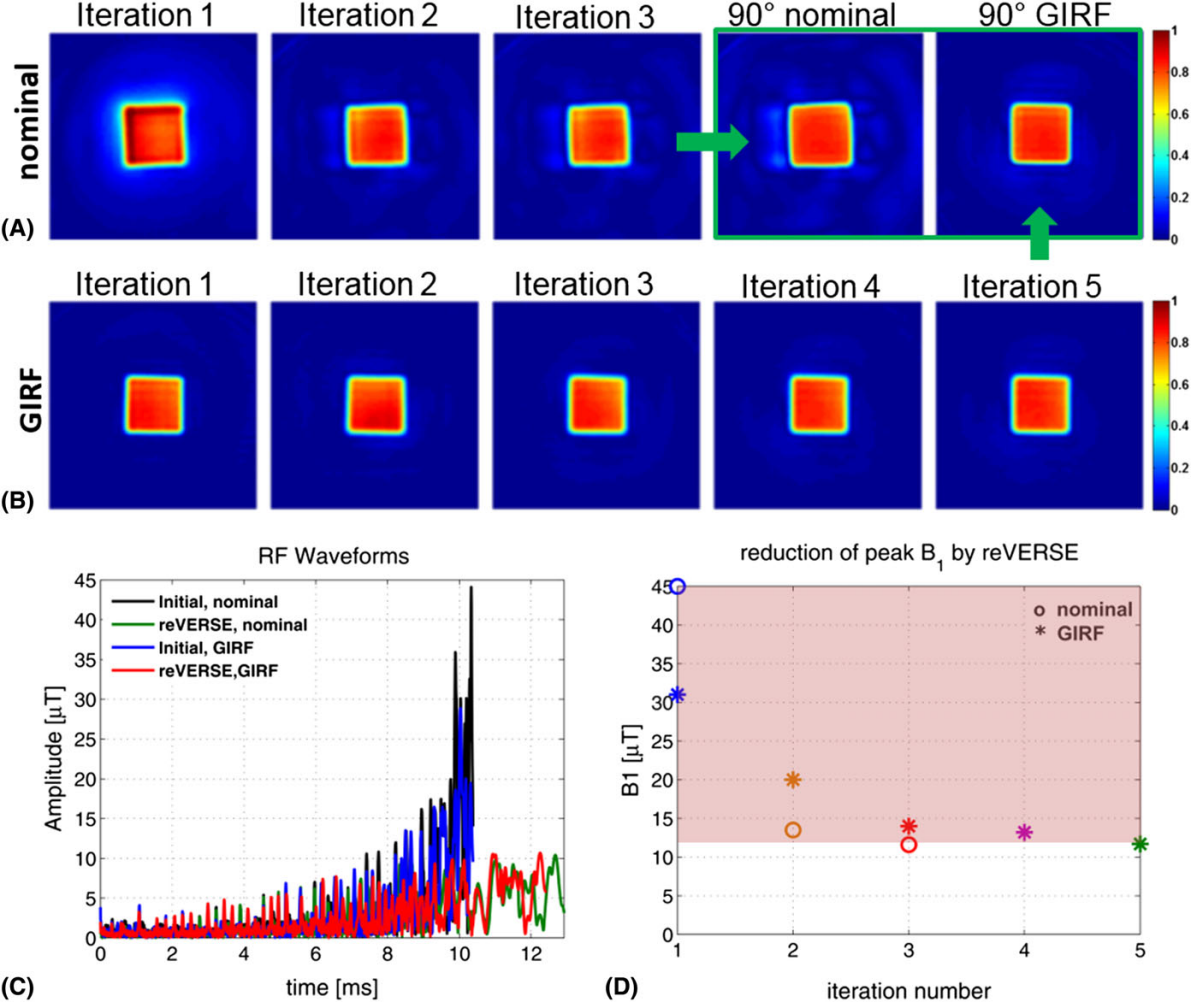
Experimental results at 3 T (Setup A) comparing excitations by reVERSE algorithm using nominal gradient waveforms (i.e. assuming ideal gradient behavior) with the proposed GIRF‐based correction. For illustration purposes, the results from each iteration of the algorithm were used to excite a square target. A, For the nominal gradients the algorithm converges more rapidly; however, experimental performance is imperfect, with distortion of the excitation and some outer‐volume signal. B, Associating with the GIRF of the system leads to more accurate excitations with reduced error across all iterations. C, The initial and reVERSEd RF waveforms designed based on the nominal and GIRF predicted gradients. D, Reduction in peak RF amplitude

Figure 6A,B depicts the results of spatially selective parallel RF excitations for the target excitation pattern in Setup A (3 T). While the reVERSE method using the nominal *k*‐space trajectory converges in three steps, integrating the GIRF predicted gradient waveforms and *k*‐space trajectories in pulse design at each iteration causes the algorithm to converge in five iterations. Table 1 summarizes the duration, peak RF magnitude and normalized root mean square error (NRMSE) values at every iteration step for nominal and GIRF predicted trajectories associated with the pulse design. The highly significant excitation error (NRMSE =56%) at the first iteration in Figure 6A quickly decreases to 18% at the third iteration. It is still, however, substantial and much higher than the results in Figure 6B. Incorporating the GIRF predicted *k*‐space trajectories into the pulse design provides significant improvement in the excitation accuracy, with NRMSE of 8%, which remains almost the same for all iterations. The results presented in the green box in Figure 6A compares the excitation results where the final RF pulses were scaled up to achieve 90° flip angle and the NRMSE is reduced from 28% to 9% by using the GIRF predicted gradients. The effect of applying VERSE on RF pulses as scaling in peak magnitude and stretching in time is clear in Figure 6C, which compares the initial and reVERSEd RF waveforms designed based on nominal and GIRF predicted gradients. The duration of the initial RF waveform increased from 10.39 ms to 12.93 ms while the peak RF amplitude was reduced from 45.12 μT to 11.38 μT for the case of nominal gradients. The final RF duration is slightly less (12.42 ms) for the case of GIRF predicted gradients where the peak RF magnitude was reduced from 30.94 μT to 11.40 μT in five iterations, as shown in Figure 6D (peak RF constraint is 12 μT).

**Table 1:** The duration, peak RF magnitude and NRMSE values at every iteration step for nominal and GIRF predicted trajectories associated with the pulse design in Setup A

Iteration	Nominal			GIRF		
	Duration [ms]	Peak [μT]	NRMSE [%]	Duration [ms]	Peak [μT]	NRMSE [%]
1	10.39	45.12	56	10.39	30.94	8
2	12.84	13.08	18	11.78	20.05	7
3	12.93	11.38	18	12.21	13.67	7
4	—	—	—	12.36	13.24	7
5	—	—	—	12.42	11.40	7

**Figure 7:**
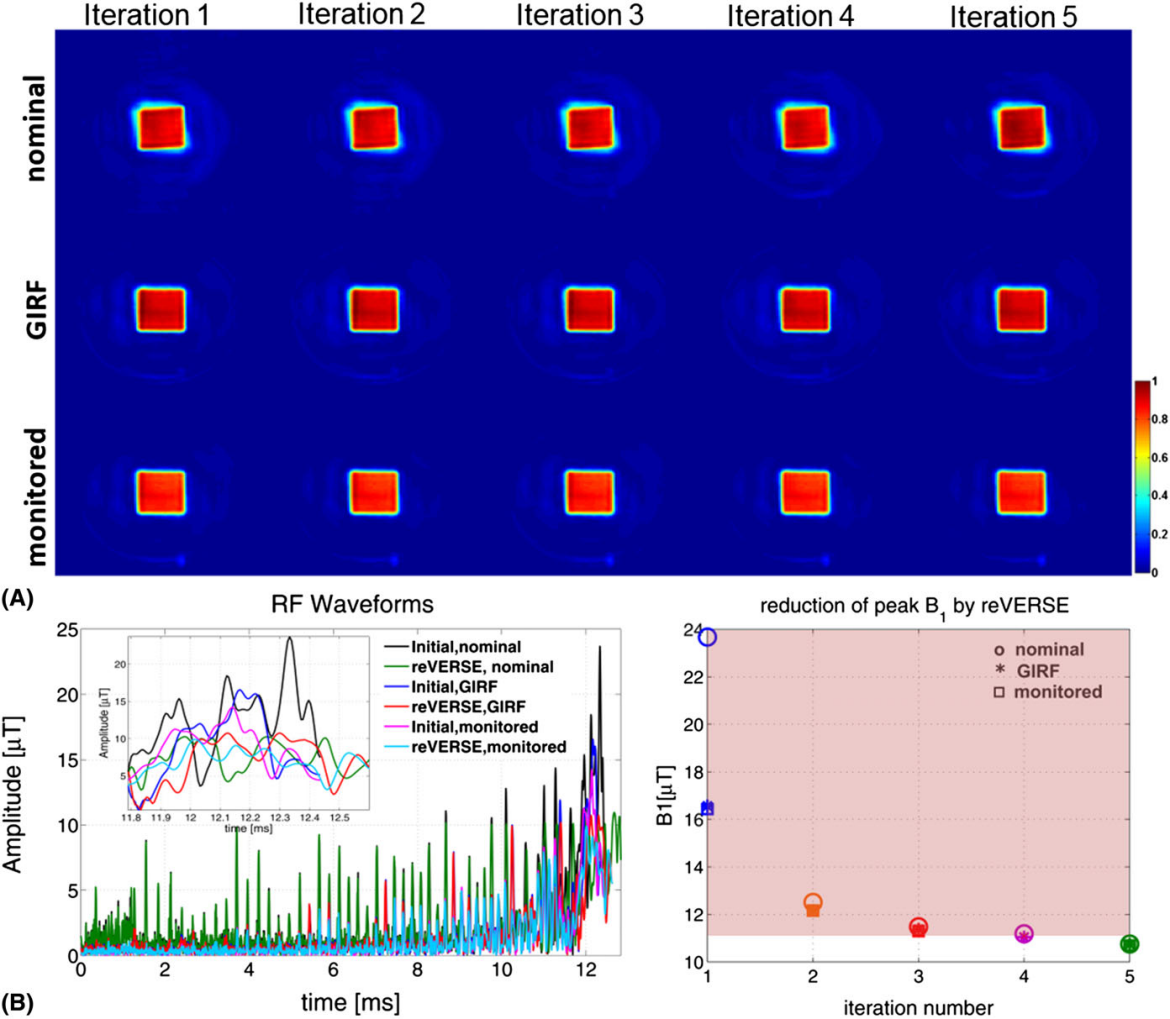
A, Experimental results at 7 T (Setup B) for the cases of nominal, GIRF predicted and monitored *k*‐space trajectories. B, The initial and reVERSEd RF pulses designed based on nominal, GIRF predicted and monitored *k*‐space trajectories. In this case the modified reVERSE method was used with GIRF predicted and directly monitored gradient waveforms. C, Reduction in peak RF amplitude

Figure 7A shows the excitation results in Setup B for the cases of nominal, GIRF predicted and monitored *k*‐space trajectories used in the pulse design to reduce the peak RF magnitude. Table 2 summarizes the duration, peak RF magnitude and NRMSE values at each iteration step for different cases. Similar to 3 T excitations, the knowledge of either GIRF predicted or directly measured *k*‐space trajectories highly improves the parallel RF excitations (i.e., NRMSE is reduced from 48% to 9% for the GIRF predicted and 8% for the directly measured gradient waveforms at the fifth iteration). While the excitation accuracies are very close to each other for GIRF predicted and monitored gradients, there is a slight difference in NRMSE up to 2%, which most likely reflects the individual deviations in multiple channel RF waveforms. Figure 7B compares the initial and reVERSEd RF pulses designed based on nominal, GIRF predicted and monitored *k*‐space trajectories. Figure 7C shows the iterative reduction of peak RF power for different cases where the peak RF constraint is selected as 11 μT.

**Table 2:** The duration, peak RF magnitude and NRMSE values at each iteration step for nominal and GIRF predicted trajectories associated with the pulse design in Setup B

Iteration	Nominal			GIRF				Monitored	
	Duration [ms]	Peak [μT]	NRMSE [%]	Duration [ms]	Peak [μT]	NRMSE [%]	Duration [ms]	Peak [μT]	NRMSE [%]
1	12.43	23.67	51	12.43	16.56	10	12.43	16.43	8
2	12.81	12.50	49	12.59	12.16	9	12.59	12.16	8
3	12.84	11.48	49	12.62	11.37	9	12.62	11.30	8
4	12.85	11.18	49	12.63	11.04	9	12.63	11.09	8
5	12.85	10.75	48	12.64	10.73	9	12.64	10.69	8

**Figure 8:**
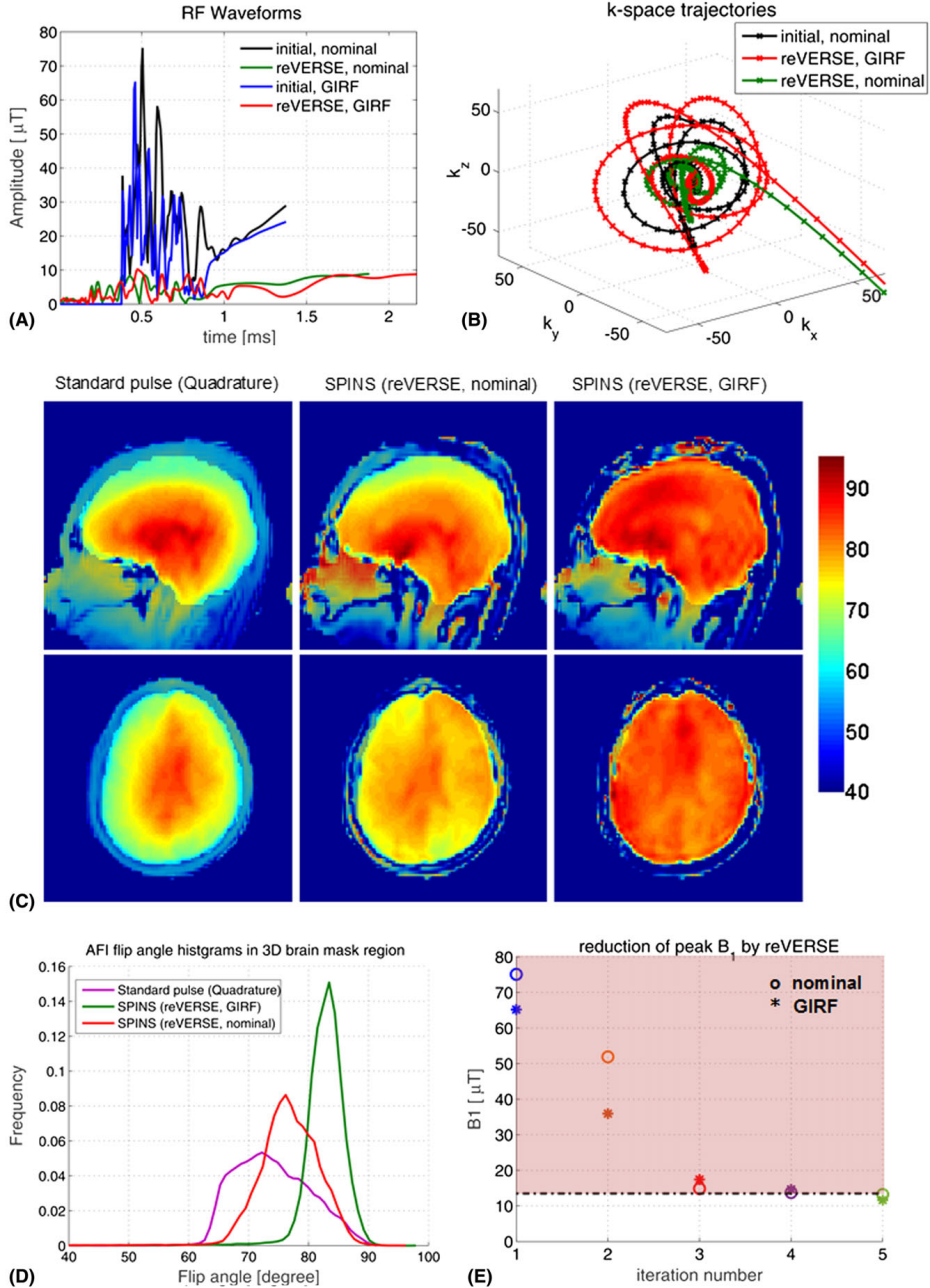
A, Initial and reVERSEd SPINS pulses. B, Nominal and GIRF predicted *k*‐space trajectories. C, *In vivo* AFI flip‐angle maps using SPINS pulses designed with and without the GIRF correction (at the fifth iteration) and the hard‐pulse excitation at the quadrature mode of the transmit array. D, Histograms of the measured flip angles within the brain. E, Reduction in peak RF amplitude

Figure 8A shows the initial and reVERSEd SPINS pulses based on the nominal and GIRF predicted *k*‐space trajectories (Figure 8B) designed for *in vivo* experiments. By applying the reVERSE algorithm, the duration of the initial RF waveform increased from 1.37 ms to 1.88 ms while the peak RF amplitude was reduced from 75.07 μT to 8.57 μT for the case of nominal gradients. The final RF duration is slightly higher (2.17 ms) for the case of GIRF predicted gradients where the peak RF magnitude was reduced from 65.18 μT to 10.2 μT in five iterations as shown in Figure 8E (peak RF constraint is 12 μT; the figure shows the peak RF values among all eight transmit channels). Figure 8C compares the acquired *in vivo* AFI flip‐angle maps using SPINS pulses designed with and without the GIRF correction (at the fifth iteration) and the hard‐pulse excitation at the quadrature mode of the transmit array. Figure 8D shows histograms of the measured flip angles within the brain. The quadrature mode excitation results a broad range of flip‐angle distribution with mean flip angle of about 72° ± 20°. The SPINS pulses computed without the GIRF correction results in a narrower range of flip angles, about 76° ± 14°, whereas the SPINS pulses computed with the GIRF correction achieve the best flip‐angle uniformity, about 83° ± 7°, besides achieving the closest excitation to the target flip angle of 90°.

## 5 DISCUSSION AND CONCLUSION

Since multidimensional parallel RF excitation techniques have been developed to control the spatial flip‐angle distribution using accelerated *k*‐space trajectories for example to overcome the RF transmit field inhomogeneity particularly in ultra‐high field MRI, the accuracy and precision demands were increased as well as the power control becoming more challenging, which has been studied by several authors.^5^, ^6^, ^12^, ^13^, ^18^, ^19^, ^20^, ^21^, ^32^ In this work, to push the experimental performance of the parallel RF excitation while keeping the applied RF power below certain thresholds by addressing the aforementioned practical challenges, we propose integrating the dynamic field monitoring and GIRF approaches into the RF pulse design, providing knowledge of the actual *k*‐space trajectories.

Due to the nature of the VERSE‐type waveform reshaping algorithms, the accuracy of both excitation and RF power limitation strictly depends on the fidelity of the local *B*_0_ fields assumed by the algorithm. One mechanism that alters the local field distribution is the off‐resonances introduced by the *B*_0_ non‐uniformities and local field susceptibilities. Lee et al.^12^ showed that such off‐resonances can cause huge excitation errors (e.g., NRMSE =142%) and corrected for this by applying an iterative reVERSE algorithm. However, they did not take into account the gradient field imperfection, another mechanism that violates the critical VERSE conditioning, which is governed by Equation (8). The performance of the VERSEd/reVERSEd pulses would degrade even more if the roughness of the reshaped gradients increases as a result of high acceleration factors in parallel transmission and aggressive RF attenuation. Furthermore, since the reVERSEd gradients are affected by the entire frequency‐dependent gradient response of the scanner, the corresponding *k*‐space trajectories will change with each iteration—a variable that is assumed to be unchanged in normal application of reVERSE (Figure 3). Our approach to address these practical challenges is to incorporate either the actual *k*‐space trajectories **k**_act_ or GIRF estimated trajectories **k**_H_ in the parallel transmit reVERSE pulse design (Figure 1). This greatly improves the multidimensional parallel excitation accuracy while achieving time optimality. Any *k*‐space trajectory can be associated and peak RF power can be controlled by setting the RF upper bound.

Using GIRFs to estimate **k**_H_ is an efficient method as a one‐time calibration procedure, because GIRFs constitute a response covering most of the deviation terms. This includes all linear distortions such as eddy currents, gradient amplifier and coil characteristics, cable effects, coil coupling and mechanical responses of the gradient system.^25^ The frequency resolution of the GIRF measurements using a dynamic field camera in Setup B is 14.3 Hz, which is fine enough even to resolve the mechanical resonances of the gradient system. An image‐based measurement method with lower frequency resolution (156 Hz) was used for experiments at 3 T, and also found to significantly improve performance.

GIRF‐based trajectory estimation is valid to the extent that the gradient system is assumed to be linear and time invariant. Field perturbations caused by non‐reproducible mechanisms (i.e. thermal drifts and sample or environment induced fields) cannot be represented by the GIRFs, thus setting an intrinsic limit to the accuracy of the estimation. Direct measurement of the actual waveforms by spatio‐temporal field monitoring provides all dynamics and full effects of the extrinsic fields that are relevant for the evolution of RF encoding. This approach requires additional field monitoring hardware, and deploying the NMR field probes and their wirings inside the RF coil may interact with the produced fields, which may explain part of the existing excitation errors. Conversely, since reVERSE is an iterative procedure, direct measurement of waveforms at each iteration of the algorithm is cumbersome. The GIRF‐based approach has the clear advantage that after the GIRF has been characterized the method can run normally on a computer with no exchange of information (or required data acquisition) on the MRI system.

Utilization of both **k**_H_ and **k**_act_ in reVERSE pulse design provides highly improved experimental performance. In Setup B the excitations based on GIRF and monitored trajectories (Figure 7A) and their NRMSE are close to each other; in the monitored case it is slightly less (1–2%), most likely due to the non‐linear field deviation terms that are not captured by GIRF. Another reason for the remaining excitation error is the fluctuation in the multiple channel RF fields. Effective RF fields also deviate from the nominal pulse shapes due to the physical limitations of the RF power amplifiers such as non‐linearity and memory effects. Concurrent RF and gradient field monitoring technology, which provides simultaneous measurements of all fields that are involved in spin excitation, can be employed to correct additionally for the RF field deviations.^40^

The *in vivo* results illustrate that, without using the GIRF to correct for non‐ideal gradients, the SPINS pulses, which are designed to create a uniform excitation, do not perform as expected. Taking into account the variable *k*‐space trajectory by associating the GIRF approach significantly improves the excitation accuracy measured in terms of flip‐angle uniformity. Both SPINS pulses as well as the quadrature mode pulse did not reach the desired mean flip angle of 90°, suggesting either that the scaling of the transmitted pulse was insufficient, or that AFI is underestimating the achieved flip angles in this high flip‐angle regime. Nevertheless, the SPINS pulse produces clearly the best excitation uniformity when used with GIRF correction, besides achieving the closest excitation to the target flip angle of 90°.

Inclusion of **k**_H_/**k**_act_ does affect the convergence and final properties of the solution obtained. A key difference from other VERSE approaches is that the *k*‐space trajectory, which is usually assumed to be constant, in fact changes through the iterations. As a result, the algorithm may sometimes converge more slowly. Further, the resulting RF pulse durations can sometimes end up longer when using the GIRF method (as with the SPINS data, Figure 8), but in other cases duration may actually be reduced (phantom data, Table 1). Since the solution *k*‐space differs for the GIRF method, the duration of the final solution depends on the new *k*‐space. Clearly this is not an optimal approach, since we do not control the final *k*‐space; however, the RF pulse design compensates for this, avoiding errors as our results have shown.

Gradient predistortion^20^ is an alternative method that iteratively converges to the desired gradient waveform by adapting iterative learning control theory to the gradient estimation problem. However, this method is based on successive measurements of the gradients that are targeting to achieve a single specified waveform, whereas in the reVERSE pulse design problem the gradient waveform is updated at each iteration. Alternatively, gradient smoothing can be performed to make the gradients less vulnerable to the system imperfections—this is a simple approach but would effectively limit the achievable slew rate, and is not guaranteed to eliminate all complex distortion effects as illustrated in our measurements.
